# Serious Games for Learning Among Older Adults With Cognitive Impairment: Systematic Review and Meta-analysis

**DOI:** 10.2196/43607

**Published:** 2023-04-12

**Authors:** Alaa Abd-alrazaq, Israa Abuelezz, Rawan AlSaad, Eiman Al-Jafar, Arfan Ahmed, Sarah Aziz, Abdulqadir Nashwan, Javaid Sheikh

**Affiliations:** 1 AI Center for Precision Health, Weill Cornell Medicine-Qatar Doha Qatar; 2 Division of Information and Computing Technology, College of Science and Engineering, Hamad Bin Khalifa University, Qatar Foundation Doha Qatar; 3 College of Computing and Information Technology, University of Doha for Science and Technology Doha Qatar; 4 Department of Health Informatics and Information Management Kuwait University Kuwait Kuwait; 5 Department of Nursing, Hazm Mebaireek General Hospital, Hamad Medical Corporation Doha Qatar; 6 Department of Public Health, College of Health Sciences, QU Health, Qatar University Doha Qatar

**Keywords:** serious games, cognitive training, learning, exergames, mild cognitive impairment, Alzheimer disease, dementia, learning, systematic reviews, meta-analysis, mobile phone

## Abstract

**Background:**

Learning disabilities are among the major cognitive impairments caused by aging. Among the interventions used to improve learning among older adults are serious games, which are participative electronic games designed for purposes other than entertainment. Although some systematic reviews have examined the effectiveness of serious games on learning, they are undermined by some limitations, such as focusing on older adults without cognitive impairments, focusing on particular types of serious games, and not considering the comparator type in the analysis.

**Objective:**

This review aimed to evaluate the effectiveness of serious games on verbal and nonverbal learning among older adults with cognitive impairment.

**Methods:**

Eight electronic databases were searched to retrieve studies relevant to this systematic review and meta-analysis. Furthermore, we went through the studies that cited the included studies and screened the reference lists of the included studies and relevant reviews. Two reviewers independently checked the eligibility of the identified studies, extracted data from the included studies, and appraised their risk of bias and the quality of the evidence. The results of the included studies were summarized using a narrative synthesis or meta-analysis, as appropriate.

**Results:**

Of the 559 citations retrieved, 11 (2%) randomized controlled trials (RCTs) ultimately met all eligibility criteria for this review. A meta-analysis of 45% (5/11) of the RCTs revealed that serious games are effective in improving verbal learning among older adults with cognitive impairment in comparison with no or sham interventions (*P*=.04), and serious games do not have a different effect on verbal learning between patients with mild cognitive impairment and those with Alzheimer disease (*P*=.89). A meta-analysis of 18% (2/11) of the RCTs revealed that serious games are as effective as conventional exercises in promoting verbal learning (*P*=.98). We also found that serious games outperformed no or sham interventions (4/11, 36%; *P*=.03) and conventional cognitive training (2/11, 18%; *P*<.001) in enhancing nonverbal learning.

**Conclusions:**

Serious games have the potential to enhance verbal and nonverbal learning among older adults with cognitive impairment. However, our findings remain inconclusive because of the low quality of evidence, the small sample size in most of the meta-analyzed studies (6/8, 75%), and the paucity of studies included in the meta-analyses. Thus, until further convincing proof of their effectiveness is offered, serious games should be used to supplement current interventions for verbal and nonverbal learning rather than replace them entirely. Further studies are needed to compare serious games with conventional cognitive training and conventional exercises, as well as different types of serious games, different platforms, different intervention periods, and different follow-up periods.

**Trial Registration:**

PROSPERO CRD42022348849; https://tinyurl.com/y6yewwfa

## Introduction

### Background

Globally, there is an aging population, that is, people are living longer, largely because of improved health care services, greater understanding of nutrition, better standard of living, and advanced biomedical research [1]. The World Health Organization estimates that the percentage of the world’s population of people aged >60 years will almost double from 12% to 22% between 2015 and 2050 [2,3]. Moreover, it is projected that the number of people aged ≥80 years will triple between 2020 and 2050, rising to 426 million [2]. However, this dramatic increase in life expectancy has not been accompanied by a proportional improvement in quality of life for older adults [4]. Generally, increased life expectancy has led to an increased risk of age-related illnesses and disabilities.

An aging population therefore grapples with various challenges, including a substantial prevalence of cognitive impairment [5], which is a decline in cognitive abilities and functions such as memory, attention, processing speed, problem-solving, language, and learning [6]. Among the top chronic diseases affecting the cognitive abilities of older adults are mild cognitive impairment (MCI) and Alzheimer disease (AD). In 2022, the Alzheimer’s Association estimated that 12% to 18% of people aged ≥60 years are living with MCI, and 10% to 15% of individuals living with MCI develop dementia each year [7]. Moreover, MCI often progresses to more severe forms of dementia such as AD [8]. Statistics have shown that 1 out of every 9 individuals in the world aged >65 years has AD [9], and it is estimated that 14 million individuals will have AD by 2050 [7]. Thus, managing and preventing age-related cognitive impairments and functions are important public health concerns.

Learning disabilities are among the major cognitive impairments in older adults. A learning disability is a condition that affects an individual’s ability to understand or use written or spoken language, perform mathematical reasoning, concentrate, or coordinate movements [10]. Learning disabilities can generally be grouped into 2 types: verbal and nonverbal. Verbal learning disorder is a learning disability that causes difficulty with basic speaking, reading, and simple social skills [11,12]. By contrast, nonverbal learning disorder is characterized by visual and spatial complications, motor problems, and difficulty understanding nonverbal information (such as body language, facial expressions, and tone of voice) [13]. Successful aging requires the ability to learn new information to be able to accomplish complex tasks. Interestingly, some types of learning seem to be relatively unaffected by normal aging, whereas others decline dramatically, but the factors that determine the extent to which learning is affected by age have not yet been fully identified. Although learning disabilities cannot be cured, they can be ameliorated through a variety of therapeutic interventions and accommodations that can make it much easier for older adults to live with learning disabilities.

Technological advances have created opportunities for the use of computerized nonpharmacological interventions, including serious games, to improve learning functions. Serious games refer to participative electronic games that are designed for goals other than entertainment, such as therapeutic rehabilitation; education; prevention of, for example, cognitive impairments; and training. Serious games have also been used to improve several cognitive functions such as memory [14], processing speed [15], executive functions [16], language processing [17], visuospatial skills [18], and global cognition [19]. Common types of serious games are exergames and cognitive training games. Exergames include physical exercises as part of the intended gameplay. By contrast, cognitive training games are video games that aim to stimulate cognitive functions such as memory, processing speed, and attention. Serious games are an emerging field of study and have recently received increased attention from researchers and practitioners as a low-cost, low-risk, enjoyable, and effective alternative to cognitive interventions [20]. Therefore, it is envisioned that serious games will have a significant potential to improve the quality of life of the aging population by supporting their learning functions, cognitive abilities, physical activity, and mental health [21,22].

### Research Gap and Aim

Many studies have assessed the effectiveness of serious games in promoting learning among older adults. The evidence from these studies has been synthesized in 2 systematic reviews; however, these reviews are undermined by the following limitations: (1) they included older adults without cognitive impairments [23], (2) they did not exclude pilot randomized controlled trials (RCTs) [23,24], (3) they did not appraise the quality of evidence [23,24], (4) they considered only particular types of serious games such as exergames [23] or cognitive training games [24], (5) they have been outdated because they were published in 2017 [23,24], and (6) they did not analyze the results of the studies according to the type of comparator (eg, no intervention, conventional exercises, or conventional cognitive training) [23,24]. To address the aforementioned limitations, this review aimed to assess the effectiveness of serious games on verbal and nonverbal learning among older adults with cognitive impairment. Our review focused on older adults with cognitive impairment, included only RCTs, assessed the quality of evidence, included all types of serious games, and analyzed results of studies according to the type of comparator. Our research question for this review was as follows: what is the effectiveness of serious games on verbal and nonverbal learning among older adults with cognitive impairment in comparison with that of other interventions such as conventional exercises, conventional cognitive training, and sham interventions?

## Methods

We followed the PRISMA (Preferred Reporting Items for Systematic Reviews and Meta-Analyses) guidelines (Multimedia Appendix 1) to perform and report this systematic review [25]. This review protocol was registered at PROSPERO (CRD42022348849) on July 26, 2022.

### Search Strategy

#### Search Sources

The first author searched the following databases on July 22, 2022: Google Scholar, IEEE Xplore, ACM Digital Library, Embase (via Ovid), MEDLINE (via Ovid), PsycINFO (via Ovid), CINAHL (via EBSCO), and Scopus. As Google Scholar’s search tools are not as advanced as those of other databases and because a Google Scholar search retrieves a significant number of papers that are automatically arranged according to their relevance, only the first 10 pages (ie, 100 hits) were considered. These databases were selected because they store studies from the target fields relevant to this review: health care (ie, MEDLINE, Embase, CINAHL, and PsycINFO), computer sciences (ie, IEEE Xplore and ACM Digital Library), or both fields (Scopus and Google Scholar). Finally, forward and backward reference list checking (ie, screening studies that referenced the included publications and screening of reference lists of the included publications and relevant reviews) was carried out.

#### Search Terms

We consulted 2 experts in digital mental health to create the search query. The search query included search terms related to the target population (ie, older adults with cognitive disorders), target intervention (ie, serious games), and target study design (ie, RCTs). Medical Subject Headings, truncation of the terms, and wildcards were considered in the search query when applicable. Multimedia Appendix 2 presents the search query used to search each of the 8 databases.

### Study Eligibility Criteria

Only RCTs that examined how effectively serious games can help older adults with cognitive impairment to improve their learning skills were included in this review. We specifically focused on studies that included serious games played on PCs, video game consoles (such as the Xbox and PlayStation), mobile phones, Nintendo consoles, tablet devices, portable devices, or any other sort of digital device. The primary element of intervention must be the game, which had to be played only for therapeutic purposes. Studies that combined serious games with other interventions were included, as long as the control group received the same adjacent intervention. Board games, card games, and other nondigital games, as well as serious games used for research, screening, diagnosis, or monitoring, were not considered in this review.

The population of the study had to be older persons (aged >60 years) with any type of cognitive impairment or disorder, as assessed by comparing baseline test results with established diagnostic criteria (eg, Mini-Mental State Examination). This review did not include studies on older people without cognitive impairment, health experts, or care providers. However, there were no restrictions on the sex and ethnicity of the participants.

The review focused on learning abilities as an outcome. Regarding the outcome measures, no restrictions were applied. Studies that simply examined acceptability, feasibility, satisfaction, or cognitive abilities other than learning were not included in this study. This review focuses on postintervention data (ie, outcome data collected immediately after the intervention). The review does not focus on follow-up data (ie, outcome data collected after a certain period had elapsed after the intervention ended) because follow-up periods were different among studies, and some studies did not follow up with participants.

This review included all types of RCTs (eg, parallel, cluster, crossover, and factorial), whereas pilot RCTs, observational studies, quasi-experiments, and reviews were not considered. Journal articles, conference proceedings, and dissertations were included. However, abstracts, conference posters, commentaries, proposals, and editorials were excluded. Studies published after 2010 in English were considered for this review. There were no restrictions on the country of publication, comparator, and research settings.

### Study Selection

We used the following procedure to find relevant studies. First, all retrieved studies were originally imported into EndNote X9 (Clarivate) to identify and eliminate duplicates. Second, 2 reviewers independently examined the titles and abstracts of all retrieved studies (the first and second authors). Third and last, both reviewers separately read the full texts of the studies that were included during the previous phase. The 2 reviewers discussed any disagreements to resolve them.

### Data Extraction

Using Microsoft Excel, 2 reviewers independently extracted data from the included studies. Before extracting the data, we pilot-tested the data extraction form using 2 (18%) of the included 11 studies. Discussions between the 2 reviewers resolved all disagreements. The data extraction form used to collect data from the included studies is shown in Multimedia Appendix 3. If the published study did not include metrics such as mean, SD, and sample size, the first and corresponding authors were contacted to obtain the data.

### Risk-of-Bias Appraisal

Using the Cochrane Risk-of-Bias 2 tool, 2 reviewers (the first and second authors) independently assessed the risk of bias in the included studies [26]. The randomization procedure, deviation from the intended intervention, missing outcome data, assessment of the outcome, and selection of the reported results are the 5 areas of RCTs where this tool assesses the risk of bias [26]. Disagreements between the reviewers regarding *risk-of-bias* judgments were resolved through discussion.

### Data Synthesis

The data obtained from the included studies were summarized using both narrative and statistical techniques. To be more precise, narrative synthesis was used to describe the study metadata, intervention features, participants, comparisons, and outcome measures using text and tables. The results of the included studies were divided into 2 groups based on the measured outcomes (ie, verbal learning and nonverbal learning). For each outcome, the results were assembled and grouped according to the comparator, which included no or sham intervention, conventional exercises, conventional cognitive training, and other serious games. Meta-analyses were conducted using Review Manager (version 5.4; The Cochrane Collaboration) when ≥2 studies from the same comparator contributed sufficient data (ie, number of participants in each intervention group, mean, and SD). The standardized mean difference (SMD; Cohen *d*) was used to determine the overall effect of each study because the outcome of interest (learning) was based on continuous data, and the included studies used various instruments to measure the outcome. Because of the significant clinical heterogeneity of the meta-analyzed studies in terms of serious game attributes (such as type, duration, frequency, and period), population characteristics (such as sample size, mean age, and health condition), and outcome measures (such as tools and follow-up periods), we decided to use the *random effects* model in the analysis.

We calculated *I*^2^ and a chi-square *P* value to evaluate the level of heterogeneity and statistical significance of heterogeneity in the meta-analyzed studies, respectively. A chi-square *P* value of ≤.05 indicates heterogeneous meta-analyzed studies [27]. The degree of heterogeneity was considered insignificant, moderate, substantial, or considerable when *I*^2^ varied from 0% to 40%, 30% to 60%, 50% to 90%, and 75% to 100%, respectively [27].

When a statistically significant difference between groups was found in a meta-analysis, we checked whether this difference was clinically significant. The term *minimal clinically important difference* (MCID) refers to the smallest change in a measured outcome that a patient would consider worthwhile and significant enough to merit a change in treatment. The MCID bounds were computed as ±0.5 times the SMD of the meta-analyzed studies.

To determine the overall quality of the evidence derived from the meta-analyses, we used the Grading of Recommendations Assessment, Development, and Evaluation approach [28], which evaluates the quality of the evidence based on 5 aspects: publication bias, indirectness, imprecision, inconsistency (ie, heterogeneity), and risk of bias [28]. Two reviewers independently evaluated the quality of the meta-analyzed data (the first and second authors). Any disagreements between the 2 reviewers were resolved by discussion. The reviewers’ interrater agreement (Cohen *κ*) was 0.89.

## Results

### Study Selection

**Figure 1:**
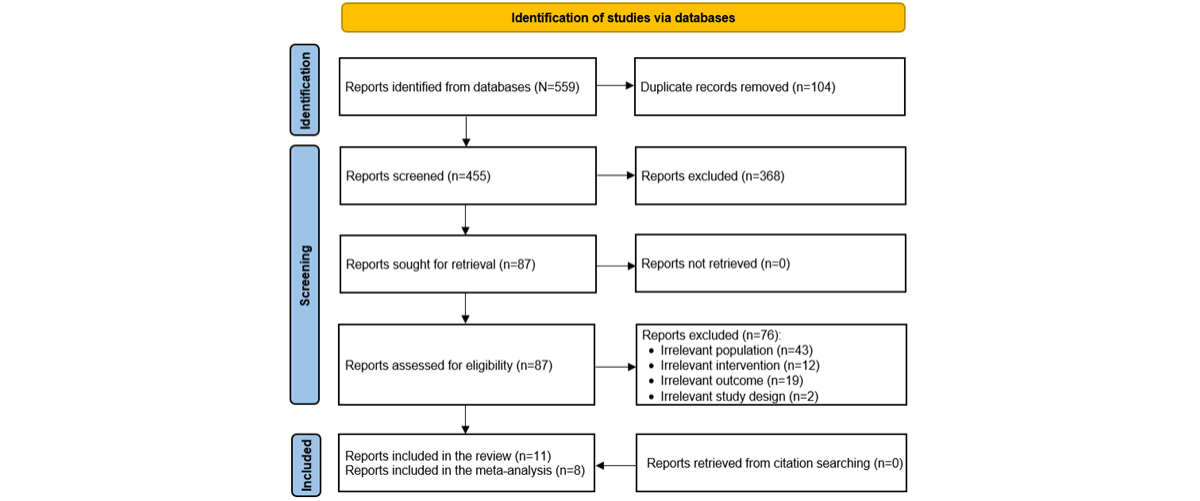
Flowchart of the study selection process.

A total of 559 records were retrieved after searching the predefined databases (Figure 1). Using EndNote X9, from these 559 records, we eliminated 104 (18.6%) duplicates. The screening of the titles and abstracts of the remaining 455 records led to the elimination of 368 (80.9%) citations. After examining the full texts of the remaining 87 publications, we excluded 76 (87%) for various reasons (Figure 1). It is worth mentioning that interrater agreements (Cohen *κ*) for screening titles and abstracts and reading full texts were 0.93 and 0.95, respectively. Backward and forward reference list checking revealed no further studies. Therefore, of the 559 studies identified initially, we included 11 (2%) in this review [29-39]. The meta-analysis included 8 (73%) of these 11 studies [29-34,38,39].

### Characteristics of the Included Studies

The selected studies were published between 2012 and 2022, but no studies were published in 2013, 2016, and 2021 (Table 1). The included studies were conducted in 7 different countries, and the country that published the most number of studies was France (3/11, 27%). Of the 11 included studies, 10 (91%) were journal articles, and 1 (9%) was a book chapter. The majority of the included studies (10/11, 91%) used parallel RCTs. The sample size in the included studies ranged from 20 to 114 participants, with an average of 56.7 (SD 33.9) participants. The mean age of the participants in the included studies was 74 (SD 4.21; range 66-81.2) years. The percentage of men in the included studies ranged from 30% to 71%, with an average of 49.2% (SD 14.5%). The mean Mini-Mental State Examination score was reported in 8 (73%) of the 11 studies, with a range of 17.9 to 28.1 and an average of 24.3 (SD 3.2). MCI was the most common disorder among participants in the included studies (8/11, 73%). Of the 11 studies, 9 (82%) recruited participants from clinical settings, whereas the remaining 2 (18%) studies recruited participants from the community.

Eleven different serious games were identified in the RCTs (Table 2). Two types of serious games were identified based on the therapeutic modality they provide: cognitive training games (10/11, 91%) and exergames (1/11, 9%). In 10 (91%) of the 11 studies, games were created with a *serious* specific goal from the outset (designed serious games). By contrast, a game in 1 (9%) of the 11 studies was not intended to be a serious game from the beginning, but it was modified to be used for a serious purpose (modified serious game). Computers were the most common platform used for playing games in the included studies (9/11, 82%). In most of the studies (8/11, 73%), serious games were played under the supervision of health professionals or caregivers. The game duration in the RCTs ranged from 7 to 90 minutes. The frequency of playing the games ranged from 2 to 5 times a week, whereas in more than half of the RCTs (6/11, 55%), it was 2 times a week. The intervention period varied from 2 to 25 weeks, but it was ≤12 weeks in 73% (8/11) of the studies.

The comparison groups received no or sham interventions in 7 (64%) of the 11 studies, whereas the groups received active interventions in 8 (73%) of the 11 studies (such as conventional exercises and other serious games; Table 3). The numbers do not add up because both active and passive comparators were used in 4 (36%) of the 11 studies. The active comparators lasted between 7 and 100 minutes. The active comparators were used 2 to 5 times a week. The active comparator period varied from 2 to 25 weeks. Of the 18 different tools used to evaluate the outcome of interest (ie, learning), the Rey-Osterrieth Complex Figure Test was most used by the included studies (4/11, 36%). The measured outcome was verbal learning in 9 (82%) of the 11 studies, whereas it was nonverbal learning in 8 (73%) of the 11 studies. The numbers do not add up because the outcomes in 6 (55%) of the 11 studies were both verbal and nonverbal. Although the outcomes were assessed immediately after the intervention in all included studies, only 4 (36%) of the 11 studies had a follow-up period, which varied from 4 to 74 weeks. Of the 11 studies, 9 (82%) reported participant attrition, which ranged from 0 to 22 (0%-22.9%) participants.

**Table 1:** Characteristics of studies and population.

Study	Country	Publication type	RCT^a^ type	Sample size, N	Age (years), mean	Male participants (%)	MMSE^b^ score	Health condition	Setting
Finn and McDonald [29]	Australia	Journal article	Parallel	31	75.6	71	28.1	MCI^c^	Clinical
Singh et al [30]	Australia	Journal article	Factorial	100	70.1	32	27.0	MCI	Community
Liu et al [31]	Taiwan	Journal article	Parallel	54	73.7	30	25.8	MCI	Community
Yang and Kwak [32]	South Korea	Journal article	Parallel	20	71.0	70	23.1	AD^d^	Clinical
Tarnanas et al [33]	Greece	Book chapter	Parallel	114	70.3	39	26.4	MCI	Clinical
Boller et al [34]	France	Journal article	Parallel	36	81.2	36.1	24.9	AD	Clinical
Flak et al [35]	Norway	Journal article	Parallel	85	66.0	66.7	NR^e^	MCI	Clinical
Gooding et al [36]	United States	Journal article	Parallel	96	75.6	58.1	NR	MCI	Clinical
Lee et al [37]	South Korea	Journal article	Parallel	20	74.3	40	17.9	AD, MCI, and dementia	Clinical
Robert et al [38]	France	Journal article	Parallel	46	79.4	47.8	21.4	Neurocognitive disorders	Clinical
Herrera et al [39]	France	Journal article	Parallel	22	76.6	50	NR	MCI	Clinical

^a^RCT: randomized controlled trial.

^b^MMSE: Mini-Mental State Examination.

^c^MCI: mild cognitive impairment.

^d^AD: Alzheimer disease.

^e^NR: not reported.

**Table 2:** Characteristics of interventions.

Study	Serious game name	Serious game type	Serious game genre	Platform	Supervision	Duration (minutes)	Frequency (per week)	Period (weeks)
Finn and McDonald [29]	E-Prime	Cognitive training game	Designed	PC	Supervised	NR^a^	2	4
Singh et al [30]	COGPACK	Cognitive training game	Designed	PC	Supervised	75	2	25
Liu et al [31]	LongGood	Exergame	Modified	Kinect	Supervised	50	3	12
Yang and Kwak [32]	Brain-Care	Cognitive training game	Designed	PC	Unsupervised	60	2	12
Tarnanas et al [33]	Virtual Reality Museum	Cognitive training game	Designed	Virtual reality headset	Supervised	90	2	21
Boller et al [34]	NR	Cognitive training game	Designed	PC	Supervised	7 to 10	3	2
Flak et al [35]	Cogmed	Cognitive training game	Designed	PC	Unsupervised	30 to 40	5	5
Gooding et al [36]	BrainFitness	Cognitive training game	Designed	PC	Both	60	2	17
Lee et al [37]	Bettercog and COMCOG	Cognitive training game	Designed	PC	Supervised	30	4	3
Robert et al [38]	MeMo	Cognitive training game	Designed	PC and tablet device	Unsupervised	30	4	12
Herrera et al [39]	NR	Cognitive training game	Designed	PC	Supervised	60	2	12

^a^NR: not reported.

**Table 3:** Characteristics of comparators and outcomes.

Study	Comparator	Duration (minutes)	Frequency (per week)	Period (weeks)	Measured outcomes	Outcome measures	Follow-up	Attrition, n
Finn and McDonald [29]	Control	N/A^a^	N/A	N/A	Verbal	WMS-IV-VPA-I^b^	After the intervention	7
Singh et al [30]	Control and conventional exercises	100	2	25	Verbal	WMS-III-LM-I^c^	After the intervention and 74-week follow-up	14
Liu et al [31]	Control and conventional exercises	50	3	12	Verbal	CVLT-DR^d^	After the intervention	4
Yang and Kwak [32]	Control	N/A	N/A	N/A	Verbal and nonverbal	ROCFT-IR^e^ and SVLT^f^	After the intervention	0
Tarnanas et al [33]	Control and conventional cognitive training	90	2	21	Verbal and nonverbal	RAVLT^g^ and ROCFT-IR	After the intervention	9
Boller et al [34]	Control and serious games	7-10	3	2	Verbal and nonverbal	FCRT^h^ and DMS48^i^	After the intervention	0
Flak et al [35]	Serious games	30-40	5	5	Verbal and nonverbal	ROCFT-IR, WMS-III-F-I^j^, WMS-III-LM-I, CVLT-II-TL^k^, and CVLT-II-SDFR^l^	4-week follow-up and 16-week follow-up	17
Gooding et al [36]	Serious games	60	2	17	Verbal and nonverbal	BSRT^m^, WMS-R-LM-I^n^, WMS-R-VR-I^o^, and WMS-R-VR-II^p^	After the intervention	22
Lee et al [37]	Serious games	30	4	3	Verbal and nonverbal	ROCFT-IR and SVLT	After the intervention	1
Robert et al [38]	Control	N/A	N/A	N/A	Nonverbal	FCSRT^q^	After the intervention and 12-week follow-up	NR^r^
Herrera et al [39]	Conventional cognitive training	60	2	12	Nonverbal	DMS48 and FCSRT	After the intervention and 24-week follow-up	NR

^a^N/A: not applicable.

^b^WMS-IV-VPA-I: Wechsler Memory Scale Fourth Edition-Verbal Paired Associates I.

^c^WMS-III-LM-I: Wechsler Memory Scale Third Edition-Logical Memory I.

^d^CVLT-DR: California Verbal Learning Test-Delayed Recall.

^e^ROCFT-IR: Rey-Osterreith Complex Figure Test-Immediate Recall.

^f^SVLT: Seoul Verbal Learning Test.

^g^RAVLT: Rey Auditory Verbal Learning Test.

^h^FCRT: Free and Cued Recall Test.

^i^DMS48: Delayed Matching-to-Sample Task, 48 items.

^j^WMS-III-F-I: Wechsler Memory Scale Third Edition-Faces I.

^k^CVLT-II-TL: California Verbal Learning Test II-Total Learning.

^l^CVLT-II-SDFR: California Verbal Learning Test II-Short Delay Free Recall.

^m^BSRT: Buschke Selective Reminding Test.

^n^WMS-R-LM-I: Wechsler Memory Scale-Revised-Logical Memory I.

^o^WMS-R-VR-I: Wechsler Memory Scale-Revised-Visual Reproductions I.

^p^WMS-R-VR-II: Wechsler Memory Scale-Revised-Visual Reproductions II.

^q^FCSRT: Free and Cued Selective Reminding Test.

^r^NR: not reported.

### Results of the Risk-of-Bias Appraisal

**Figure 2:**
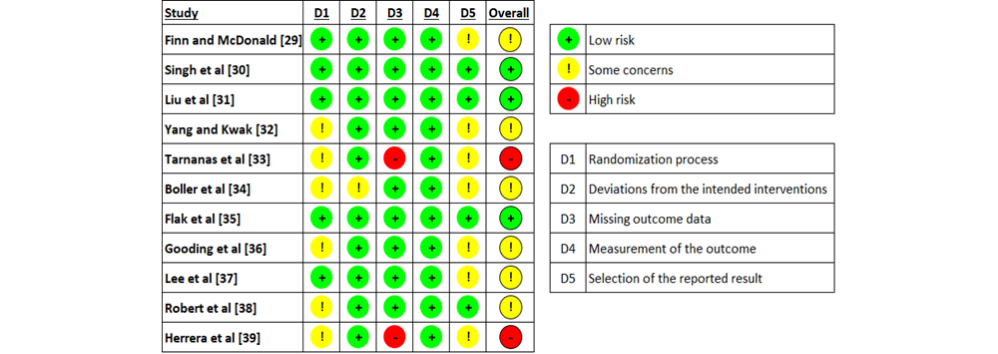
Reviewers’ judgments about each risk-of-bias domain for each included study.

**Figure 3:**
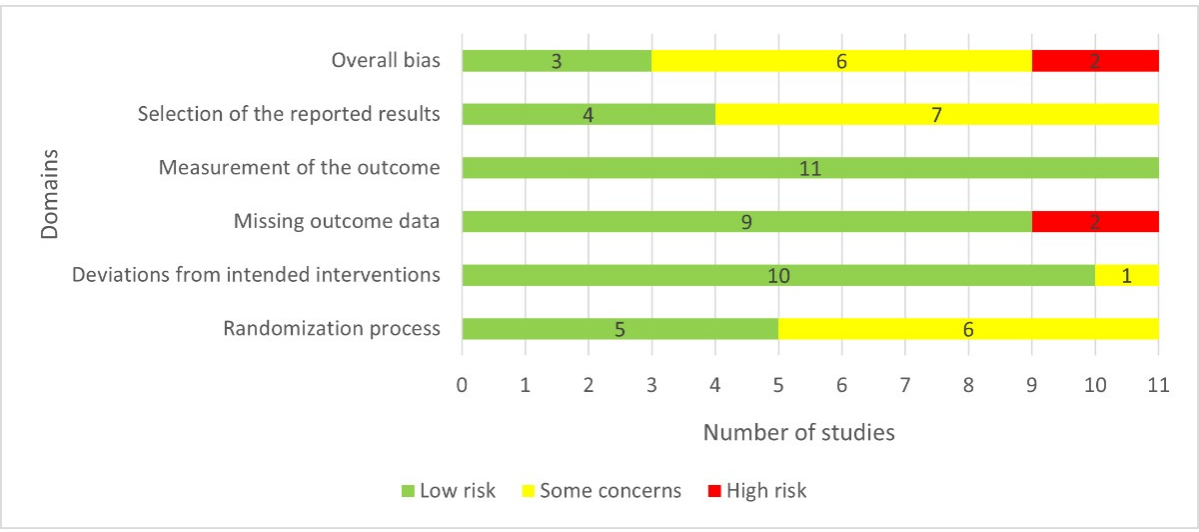
Reviewers’ judgments about each risk-of-bias domain.

Reviewers’ judgments about each *risk-of-bias* domain for each included study are presented in Figure 2. A low risk of bias in the *randomization process* domain was observed in 45% (5/11) of the studies (Figure 3). The majority of the studies were judged to have a low risk of bias in 2 domains: deviations from the intended intervention (10/11, 91%) and missing outcome data (9/11, 82%). The risk of bias in the measurement of the outcome was low in all studies. In 4 (36%) of the 11 studies, the risk of bias in the selection of the reported results was low. According to these judgments, only 3 (27%) of the 11 studies were judged to have a low risk of bias in the last domain (ie, overall bias). The interrater agreement (Cohen *κ*) between the reviewers in *risk-of-bias* judgments was 0.87.

### Results of the Studies

#### Verbal Learning

##### Overview

The effectiveness of serious games on verbal learning was assessed in 9 (82%) of the 11 studies [29-37]. The comparators in these studies were no or sham interventions, conventional cognitive training, conventional exercises, and other serious games. Accordingly, the results of the studies were grouped based on these comparators.

##### Serious Games Versus No or Sham Interventions

**Figure 4:**
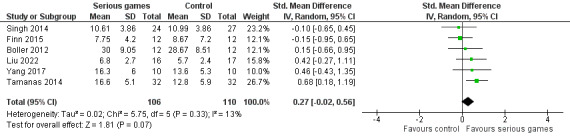
Forest plot of 6 studies comparing the effectiveness of serious games with that of no or sham interventions on verbal learning.

The effects of serious games and no or sham interventions on verbal learning were compared in more than half of the studies (6/11, 55%) [29-34]. As shown in Figure 4, a meta-analysis of the results of these studies showed no statistically significant difference (*P*=.07) in verbal learning between the serious game group and the control group (SMD 0.27, 95% CI –0.02 to 0.56). The statistical heterogeneity of the evidence was not a concern (*P*=.33; *I*^2^=13%). The quality of the evidence was very low because it was downgraded by 3 levels owing to a high risk of bias and imprecision (Multimedia Appendix 4).

**Figure 5:**
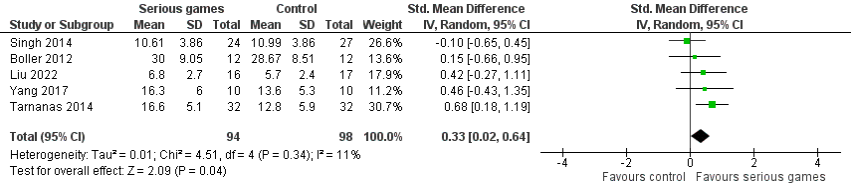
Forest plot of 5 studies comparing the effectiveness of serious games with that of no or sham interventions on verbal learning.

We conducted a sensitivity analysis by removing 1 (13%) of the 8 meta-analyzed studies [29]. The study was removed because its control group had higher verbal learning at baseline (before the intervention) than its serious game group (6.5 vs 5.4), and the pre- and postintervention changes in verbal learning were slightly higher for the serious game group than for the control group (2.33 vs 2.17); hence, using only the postintervention results of this study would be inappropriate. As presented in Figure 5, the sensitivity analysis showed a statistically significant difference (*P*=.04) in verbal learning between the groups, favoring serious games over no or sham interventions (SMD 0.33, 95% CI 0.02-0.64). This difference was also clinically important because the overall effect was outside the MCID boundaries (−0.165 to 0.165), and its CI did not cross the *no-effect* line (zero effect). The heterogeneity of the evidence remained statistically insignificant (*P*=.34; *I*^2^=11%). The quality of the evidence was very low because it was downgraded by 3 levels owing to a high risk of bias, heterogeneity, and imprecision (Multimedia Appendix 4).

**Figure 6:**
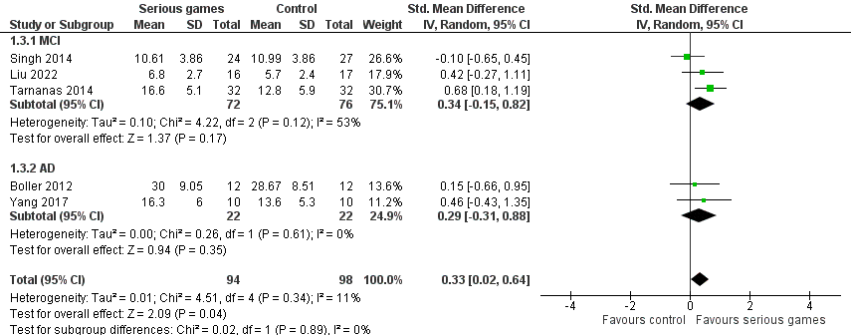
Forest plot of 5 studies comparing the effectiveness of serious games on older adults with mild cognitive impairment (MCI) with that on older adults with Alzheimer disease (AD).

Given that participants had AD in 2 (25%) of the 8 meta-analyzed studies [32,34] and MCI in 3 (38%) of the 8 studies [30,31,33], a subgroup analysis was carried out to check whether serious games have a different effect on verbal learning among patients with different diseases (MCI vs AD). The subgroup analysis showed no statistically significant difference (*P*=.89) in the effects of serious games on verbal learning between patients with MCI and those with AD (Figure 6).

##### Serious Games Versus Conventional Exercises

**Figure 7:**
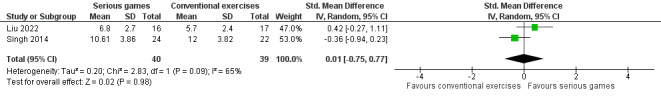
Forest plot of 2 studies comparing the effectiveness of serious games with that of conventional exercises on verbal learning.

The effectiveness of serious games on verbal learning was compared with that of conventional exercises in only 2 (18%) of the 11 studies [30,31]. As depicted in Figure 7, a meta-analysis of the results of these studies showed no statistically significant difference (*P*=.98) in verbal learning between the serious game group and the conventional exercises group (SMD 0.01, 95% CI –0.75 to 0.77). The statistical heterogeneity of the evidence was substantial (*P*=.09; *I*^2^=65%). The quality of the evidence was very low because it was downgraded by 4 levels owing to high heterogeneity and imprecision (Multimedia Appendix 4).

##### Serious Games Versus Other Serious Games

The effectiveness of serious games on verbal learning was compared with that of other serious games in 4 (36%) of the 11 studies [34-37]. The results of these studies were not synthesized statistically in this review because the serious games in the comparison groups in these studies were dissimilar. In the study by Lee et al [37], a cognitive training game (COMCOG) that focuses on improving only memory and attention was compared with another cognitive training game (Bettercog) that targets various cognitive abilities (ie, orientation, attention, memory, language, executive function, visuospatial function, calculation, and motor functions). This study showed no statistically significant difference in verbal learning between the COMCOG and Bettercog groups [37]. In the study by Flak et al [35], a serious game that adjusts the level of difficulty of the tasks based on the individual’s mastery at each level (ie, adaptive game) was compared with the same game but without adjustment of the level of difficulty of the tasks (ie, nonadaptive game). The study found no statistically significant difference in verbal learning between the adaptive game group and the nonadaptive game group [35].

The study by Gooding et al [36] compared the effects of 3 serious games on verbal learning: (1) an empirically validated game with an incorporated motivational therapeutic milieu based on the principles put forth by the neuropsychological and educational approach to remediation model of treatment (BrainFitnessPlus), (2) the same aforementioned game but without an incorporated motivational therapeutic milieu (BrainFitness), and (3) different commercially available computer games (ie, BrainAge, sudoku, and crossword puzzles). The study showed no statistically significant difference between the BrainFitnessPlus group and the BrainFitness group in verbal learning as measured by the Buschke Selective Reminding Test and Wechsler Memory Scale-Revised-Logical Memory I [36]. However, BrainFitnessPlus performed better than the commercially available computer games on verbal learning as measured by the Buschke Selective Reminding Test, and BrainFitness performed better than the commercially available computer games on verbal learning as measured by the Wechsler Memory Scale-Revised-Logical Memory I [36]. In the study by Boller et al [34], the effects of 2 cognitive training games on nonverbal learning were assessed. Both games consisted of a study phase and a test phase. In each session, players of both games had to read and remember 16 words presented one at a time on a computer screen for 3 seconds, followed by a white screen lasting 1 second [34]. In the test phase, the players were asked to recognize the 16 study words, which were mixed with 16 new words in the first game (recollection training game) and 32 new words in the second game (recognition practice game) [34]. The study showed no statistically significant difference in nonverbal learning between the 2 groups [34].

##### Serious Games Versus Conventional Cognitive Training

The effectiveness of serious games on verbal learning was compared with that of conventional cognitive training in only 1 (9%) of the 11 RCTs [33]. The study showed no statistically significant difference in verbal learning between the serious game group and the conventional cognitive training group [33].

#### Nonverbal Learning

##### Overview

The effectiveness of serious games on nonverbal learning was assessed in 8 (73%) of the 11 studies [32-39]. Similar to verbal learning studies, the comparators in these studies were no or sham interventions, conventional cognitive training, conventional exercises, and other serious games. Accordingly, the results of the studies were grouped based on these comparators.

##### Serious Games Versus No or Sham Interventions

**Figure 8:**
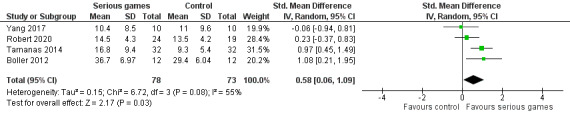
Forest plot of 4 studies comparing the effectiveness of serious games with that of no or sham interventions on nonverbal learning.

The effects of serious games and no or sham interventions on nonverbal learning were compared in 4 (36%) of the 11 studies [32-34,38]. As shown in Figure 8, a meta-analysis of the results of these studies showed a statistically significant difference (*P*=.03) in nonverbal learning between the groups, favoring serious games over no or sham interventions (SMD 0.58, 95% CI 0.06-1.09). This difference was also clinically important because the overall effect was outside the MCID boundaries (−0.29 to 0.29), and its CI did not cross the *no-effect* line (zero effect). The statistical heterogeneity of the evidence was moderate (*P*=.08; *I*^2^=55%). The quality of the evidence was very low because it was downgraded by 4 levels owing to a high risk of bias, heterogeneity, and imprecision (Multimedia Appendix 5).

##### Serious Games Versus Conventional Cognitive Training

**Figure 9:**
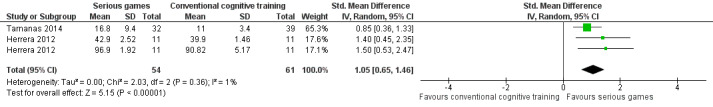
Forest plot of 2 studies comparing the effectiveness of serious games with that of conventional cognitive training on nonverbal learning.

The effectiveness of serious games on nonverbal learning was compared with that of conventional cognitive training in only 2 (18%) of the 11 studies [33,39]. One of these studies used 2 different measures to assess nonverbal learning [39]; therefore, our meta-analysis included 3 comparisons from these studies (Figure 9). The meta-analysis demonstrated a statistically significant difference (*P*<.001) in nonverbal learning between the groups, favoring serious games over conventional cognitive training (SMD 1.05, 95% CI –0.65 to 1.46). This difference was also clinically important because the overall effect was outside the MCID boundaries (−0.525 to 0.525), and its CI did not cross the *no-effect* line (zero effect). The statistical heterogeneity of the evidence was not a concern (*P*=.36; *I*^2^=1%). The quality of the evidence was low because it was downgraded by 2 levels owing to a high risk of bias (Multimedia Appendix 5).

##### Serious Games Versus Other Serious Games

Similar to verbal learning, the effectiveness of serious games on nonverbal learning was compared with the effects of other serious games in 4 (36%) of the 11 studies [34-37]. The results of these studies were not synthesized statistically in this review because the serious games in the comparison groups in these studies were dissimilar. The study by Lee at al [37] compared 2 serious games (COMCOG vs Bettercog) and showed no statistically significant difference in nonverbal learning between the groups. The study by Flak et al [35] assessed the effect of an adaptive serious game on nonverbal learning in comparison with that of a nonadaptive serious game, and it found no statistically significant difference in nonverbal learning between the groups. The study by Gooding et al [36] compared 3 games—BrainFitnessPlus, BrainFitness, and commercially available computer games—and showed no statistically significant difference in nonverbal learning among the 3 groups. The study by Boller et al [34] investigated the effect of a recollection training game on nonverbal learning in comparison with that of a recognition practice game and reported that there was a statistically significant difference in nonverbal learning between the groups, favoring the recollection training game over the recognition practice game.

## Discussion

### Principal Findings

This review assessed the effectiveness of serious games on verbal learning among older adults with cognitive impairment. Our meta-analysis revealed that serious games are effective in improving verbal learning among older adults with cognitive impairment in comparison with no or sham interventions. According to our subgroup analysis, serious games do not have a different effect on verbal learning between patients with AD and those with MCI. Our review demonstrated that serious games are as effective as conventional exercises in promoting verbal learning among older adults with cognitive impairment, and this could be attributed to 2 reasons. First, only 2 (18%) of the 11 studies were included in the relevant meta-analysis [30,31]. Second, the serious games in these studies are of different types (cognitive training games [30] and exergames [31]) and genres (designed [30] and modified [31]) and have different platforms (PC [30] and Kinect [31]). In this review, we found that serious games outperformed no or sham interventions and conventional cognitive training in enhancing nonverbal learning among older adults with cognitive impairment. It is worth mentioning that none of the findings are based on high-quality evidence.

A previous review compared the effectiveness of serious games (cognitive training games in particular) with that of any intervention (passive or active intervention) on verbal and nonverbal learning among older adults with MCI and dementia [24]. In contrast to our findings, the study showed that serious games have a different effect between patients with AD and those with dementia [24]. To be more specific, the previous review [24] demonstrated that serious games are more effective than other interventions in improving verbal learning (*P*=.002) and nonverbal learning (*P*<.001) among older adults with MCI, whereas they are as effective as other interventions in enhancing verbal learning (*P*=.14) and nonverbal learning (*P*=.50). Another review examined the effectiveness of serious games (exergames in particular) on verbal and nonverbal learning among older adults in comparison with that of any intervention (passive or active intervention) [23]. Contrary to our findings, the review showed that serious games are as effective as other interventions in enhancing verbal learning (*P*=.09) and nonverbal learning (*P*=.26). The contrary findings between our review and the previous reviews can be attributed to the main differences between them as mentioned in the Research Gap and Aim subsection under the Introduction section.

### Practical and Research Implications

Although this review demonstrated that serious games have the potential to improve verbal and nonverbal learning among older adults with cognitive impairment, our findings remain inconclusive for 3 reasons. First, the quality of evidence from most of the meta-analyses (5/6, 83%) was very low mainly because of the high risk of bias, low homogeneity, and lack of precision of the estimated total effect sizes. Second, the meta-analyses included a small number of RCTs (2-6). Third and last, most of the meta-analyzed studies (6/8, 75%) had a small sample size. Therefore, until further convincing proof of their effectiveness is offered, serious games should be used to supplement current interventions for verbal and nonverbal learning rather than replace them entirely.

Although PCs were used to deliver serious games in most of the included studies (9/11, 82%), no study used mobile devices (eg, smartphones and tablet devices), and only 1 (9%) of the 11 studies used VR headsets. As a PC allows for more distraction from outside sources, a serious game delivered through a VR headset might be able to hold the user’s attention for longer than a serious game delivered through a PC. In addition, mobile devices are more appealing because they are typically more affordable, widespread, and accessible than traditional platforms such as PCs and game consoles. In 2021, there were approximately 15 billion mobile devices and 7.1 billion mobile users worldwide. By 2025, it is anticipated that these numbers will rise significantly [40]. Therefore, game developers need to collaborate with medical experts and develop serious games that can be played on mobile devices and with VR headsets. This could pose a challenge because commercial game development is a lucrative industry and to convince game developers to develop serious games that can be played on nontraditional platforms would require incentives with appropriate funding sources. The result, however, would be commercial-level user-tested and appealing games designed especially with the aging population with cognitive impairment in mind. Given that none of the serious games in the included studies were designed specifically to improve learning, this approach could be groundbreaking.

Further research is needed to address the following limitations and gaps in the previous studies and to confirm the results with a larger evidence base: (1) lack of studies comparing serious games with other interventions such as conventional cognitive training and conventional exercises; (2) lack of studies comparing different types of serious games (adaptive vs nonadaptive games and exergames vs cognitive training games) and different platforms used to play serious games (PCs vs VR headsets vs mobile devices); (3) limited research on the effectiveness of serious games on learning among older adults with other cognitive disorders such as vascular dementia, Lewy body dementia, and Huntington disease; (4) lack of a follow-up period in most studies; (5) short intervention periods (≤3 months); (6) scarcity of studies conducted in low-income countries; (7) missing information of the mean and SD of pre- and postintervention changes in learning for each group; (8) small sample sizes; (9) issues of high risk of bias mainly in the randomization process or selection of the reported results; (10) missing information on the underlying psychological methodology or intervention of the serious games; and (11) variation in the selection of tools to assess verbal and nonverbal learning.

### Limitations

This review focused on the short-term effect of digital serious games on learning among older adults with cognitive impairment. Therefore, we cannot comment on the effectiveness of other interventions (nondigital serious games used for other purposes), on other outcomes (eg, long-term effect on memory, attention, and processing speed), or on intervention effectiveness among other populations (adolescents, young adults, middle-aged adults, and those without cognitive impairment) because they were beyond the scope of this review.

Another limitation of this review is that the effect sizes might be overestimated or underestimated, given that we used postintervention data rather than the pre- and postintervention change data to estimate the effect size for each study. Postintervention data were used because none of the meta-analyzed studies reported the mean and SD of the pre- and postintervention changes in learning, and there was no statistically significant difference in verbal and nonverbal learning between the groups at baseline in the majority of the meta-analyzed studies (7/8, 88%).

Most likely, some relevant studies were missed in this review, given that we did not include quasi-experiments, pilot RCTs, and studies published in a language other than English before 2010. There may be a concern about the internal validity of our findings because the quality of evidence in most of the meta-analyses (5/6, 83%) was very low.

### Conclusions

Serious games can play a significant role in promoting verbal and nonverbal learning among older adults with cognitive impairment. Nevertheless, our findings remain inconclusive for 3 reasons. First, the quality of evidence from most of the meta-analyses (5/6, 83%) was very low mainly because of the high risk of bias, low homogeneity, and lack of precision of the estimated total effect sizes. Second, the meta-analyses included a small number of RCTs (2-6). Third and last, most of the meta-analyzed studies (6/8, 75%) had a small sample size. Hence, until further convincing proof of their effectiveness is offered, serious games should be used to supplement current interventions for verbal and nonverbal learning rather than replace them entirely. Game developers should consider developing serious games that can be played on mobile devices and with VR headsets. Further studies should be conducted to compare (1) serious games with other interventions such as conventional cognitive training and conventional exercises, (2) different types of serious games (adaptive vs nonadaptive games and exergames vs cognitive training games), (3) different platforms used to play serious games (PCs vs VR headsets vs mobile devices), (4) different intervention periods, and (5) short-term effect with long-term effectiveness of serious games.

## Appendix

Multimedia Appendix 1 PRISMA (Preferred Reporting Items for Systematic Reviews and Meta-Analyses) checklist.

Multimedia Appendix 2 Search strategy.

Multimedia Appendix 3 Data extraction form.

Multimedia Appendix 4 Grading of Recommendations Assessment, Development, and Evaluation profile for comparison of serious games with control and conventional exercises for verbal learning.

Multimedia Appendix 5 Grading of Recommendations Assessment, Development, and Evaluation profile for comparison of serious games with control and conventional exercises for nonverbal learning.

## Abbreviations

AD: Alzheimer disease
MCI: mild cognitive impairment
MCID: minimal clinically important difference
PRISMA: Preferred Reporting Items for Systematic Reviews and Meta-Analyses
RCT: randomized controlled trial
SMD: standardized mean difference
VR: virtual reality
