# Supplementary material for: Serious Games for Learning Among Older Adults With Cognitive Impairment: Systematic Review and Meta-analysis
Source: J Med Internet Res. 2023 Apr 12;25:e43607. doi: 10.2196/43607 (PMC10134019; doi:10.2196/43607)
Supplement: Multimedia Appendix 4 [file jmir_v25i1e43607_app4.docx]

**Appendix 4: GRADE Profile for comparison of serious games to control and conventional exercises for verbal learning**

| **Certainty assessment** | | | | | | | **Summary of findings** | | | | |
| --- | --- | --- | --- | --- | --- | --- | --- | --- | --- | --- | --- |
| **Participants (studies) Follow-up** | **Risk of bias** | **Inconsistency** | **Indirectness** | **Imprecision** | **Publication bias** | **Overall certainty of evidence** | **Study event rates (%)** | | **Relative effect (95% CI)** | **Anticipated absolute effects** | |
|  |  |  |  |  |  |  | **Control** | **Serious games** |  | **Risk with** | **Risk difference with Serious games** |
| **Serious games vs. Control** | | | | | | | | | | | |
| 216 (6 RCTs) | very serious^a^ | not serious | not serious | serious^b,c^ | none | ⨁◯◯◯ Very low | 110 | 106 | - | - | SMD **0.27 higher** (0.02 lower to 0.56 higher) |
| **Serious games vs. Control (sensitivity analysis)** | | | | | | | | | | | |
| 192 (5 RCTs) | very serious^a^ | not serious | not serious | Serious^b,d^ | none | ⨁◯◯◯ Very low | 94 | 98 | - | - | SMD **0.33 higher** (0.02 higher to 0.64 higher) |
| **Serious games vs. Conventional exercises** | | | | | | | | | | | |
| 79 (2 RCTs) | not serious | very serious^e^ | not serious | very serious^f,g^ | none | ⨁◯◯◯ Very low | 39 | 40 | - | - | SMD **0.01 higher** (0.75 lower to 0.77 higher) |

**CI:** confidence interval; **SMD:** standardised mean difference

#### Explanations

a. Evidence was downgraded by 2 levels because only 2 of the meta-analyzed studies in this comparison were judged to have a low risk of bias, this is due to issues mainly in the randomization process and selection of the reported results.

b. Evidence was downgraded by 1 level because 95% CI crosses one of the two MID boundaries for this outcome.

c. MID for this outcome, calculated as ± 0.5 times the standardized mean difference (SMD), is ± 0.135

d. MID for this outcome, calculated as ± 0.5 times the standardized mean difference (SMD), is ± 0.165

e. Evidence was downgraded by 2 levels as P=0.09 and I^2^=65%, indicating high heterogeneity.

f. Evidence was downgraded by 2 levels because 95% CI crosses both MID boundaries for this outcome.

g. MID for this outcome, calculated as ± 0.5 times the standardized mean difference (SMD), is ± 0.005
