# Supplementary material for: Serious Games for Learning Among Older Adults With Cognitive Impairment: Systematic Review and Meta-analysis
Source: J Med Internet Res. 2023 Apr 12;25:e43607. doi: 10.2196/43607 (PMC10134019; doi:10.2196/43607)
Supplement: Multimedia Appendix 5 [file jmir_v25i1e43607_app5.docx]

**Appendix 5: GRADE Profile for comparison of serious games to control and conventional exercises for nonverbal learning**

| **Certainty assessment** | | | | | | | **Summary of findings** | | | | |
| --- | --- | --- | --- | --- | --- | --- | --- | --- | --- | --- | --- |
| **Participants (studies) Follow-up** | **Risk of bias** | **Inconsistency** | **Indirectness** | **Imprecision** | **Publication bias** | **Overall certainty of evidence** | **Study event rates (%)** | | **Relative effect (95% CI)** | **Anticipated absolute effects** | |
|  |  |  |  |  |  |  | **Control** | **Serious games** |  | **Risk with** | **Risk difference with Serious games** |
| **Serious games vs. Control** | | | | | | | | | | | |
| 151 (4 RCTs) | very serious^a^ | serious^b^ | not serious | serious^c,d^ | none | ⨁◯◯◯ Very low | 73 | 78 | - | - | SMD **0.58 higher** (0.06 higher to 1.09 higher) |
| **Serious games vs. Conventional cognitive activities** | | | | | | | | | | | |
| 115 (2 RCTs) (3 comparisons) | very serious^e^ | not serious | not serious | not serious | none | ⨁⨁◯◯ Low | 61 | 54 | - | - | SMD **1.05 higher** (0.65 higher to 1.46 higher) |

**CI:** confidence interval; **SMD:** standardised mean difference

#### Explanations

a. Evidence was downgraded by 2 levels because none of the meta-analyzed studies in this comparison was judged to have a low risk of bias, this is due to issues mainly in the randomization process and selection of the reported results.

b. Evidence was downgraded by 1 level as P=0.08 and I^2^=55%, indicating moderate heterogeneity.

c. Evidence was downgraded by 1 level because 95% CI crosses one of the two MID boundaries for this outcome.

d. MID for this outcome, calculated as ± 0.5 times the standardized mean difference (SMD), is ± 0.29

e. Evidence was downgraded by 2 levels because the meta-analyzed studies in this comparison were judged to have a high risk of bias, this is due to issues mainly in the randomization process, missing outcome data, and selection of the reported results.
